# Supplementary figures and images for: Type I interferon regulates proteolysis by macrophages to prevent immunopathology following viral infection
Source: PLoS Pathog. 2022 May 5;18(5):e1010471. doi: 10.1371/journal.ppat.1010471 (PMC9113601; doi:10.1371/journal.ppat.1010471)

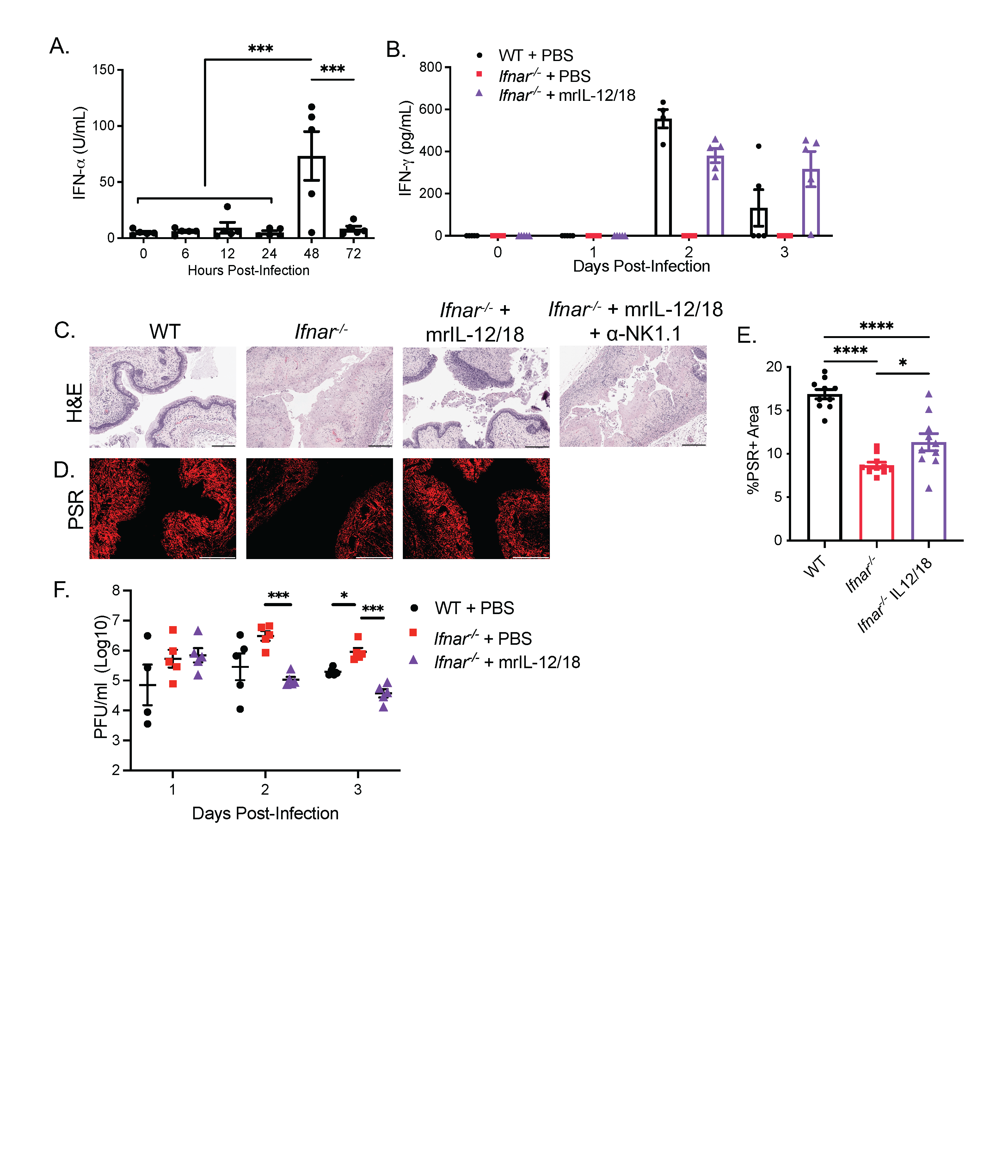

Supplement: S1 Fig — (A) IFN-α cytokine levels in vaginal washes of WT mice infected with 104 PFU of HSV-2 333 intravaginally (n = 5). (B) IFΝ-γ cytokine levels in vaginal washes of HSV-2-infected WT, Ifnar-/-, and Ifnar-/- + mrIL-12/18 mice at 0 to 3 dpi (n = 5). (C) H&E staining of vaginal cross-sections of HSV-2-infected WT, Ifnar-/- + PBS, Ifnar-/- + mrIL-12/18, and Ifnar-/- + mrIL-12/18 + α-NK1.1 at 3 dpi. (D and E) PSR staining of vaginal cross-sections (D) and quantification of % PSR+ to total vaginal area (E) of HSV-2-infected WT, Ifnar-/-, and Ifnar-/- + mrIL-12/18 at 3 dpi. (F) Viral titers in vaginal washes of HSV-2-infected WT, Ifnar-/-, and Ifnar-/- + mrIL-12/18 mice at 1 to 3 dpi. H&E scale bar represents 200 μm, PSR scale bar represents 100 μM. Data in (A), (B), (E), (F), are represented as mean ± SEM. *p < 0.05, ***p < 0.001, and ****p < 0.0001 (A, E, one-way ANOVA; B, F, two-way ANOVA). (TIF) [file ppat.1010471.s001.tif]

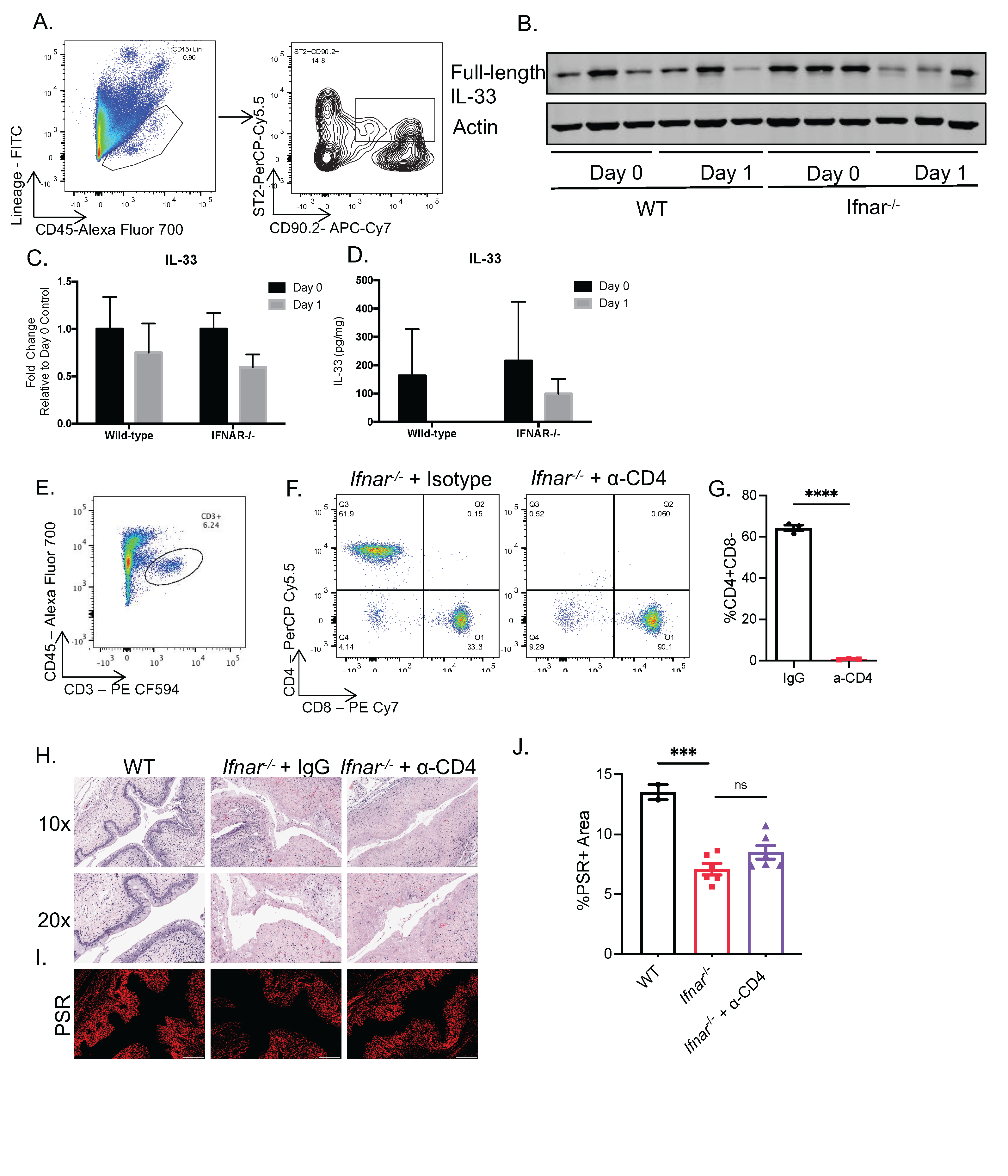

Supplement: S2 Fig — (A) Representative flow plots for CD45+Lin-ST2+CD90.2+ ILC2s in vaginal tissue of HSV-2-infected mice (B to C) Western blot (B) for IL-33 quantification as fold change (C) in vaginal tissue of HSV-2-infected WT and Ifnar-/- mice at 0 and 1 dpi. (D) IL-33 cytokine levels in vaginal washes of HSV-2-infected WT and Ifnar-/- mice at 0 and 1 dpi. (E) Representative flow plots for CD45+CD3+ T cells gating. (F and G) Representative flow plots (F) and quantification (G) for α-CD4 Ab depletion in blood of HSV-2-infected Ifnar-/- mice at 3 dpi. (H) H&E staining of vaginal cross-sections 3 dpi in WT, Ifnar-/- + IgG and Ifnar-/- + α-CD4 Ab. (I and J) PSR staining (I) of vaginal cross-sections and quantification of % PSR+ to total vaginal tissue area (J) of WT, Ifnar-/- + IgG and Ifnar-/- + α-CD4 Ab 3dpi. Data in (C), (D), (G), and (J) are represented as mean ± SEM. ***p < 0.001, and ****p < 0.0001 (C, D, two-way ANOVA; H, one-way ANOVA; J, two-tailed t-test). (TIF) [file ppat.1010471.s002.tif]

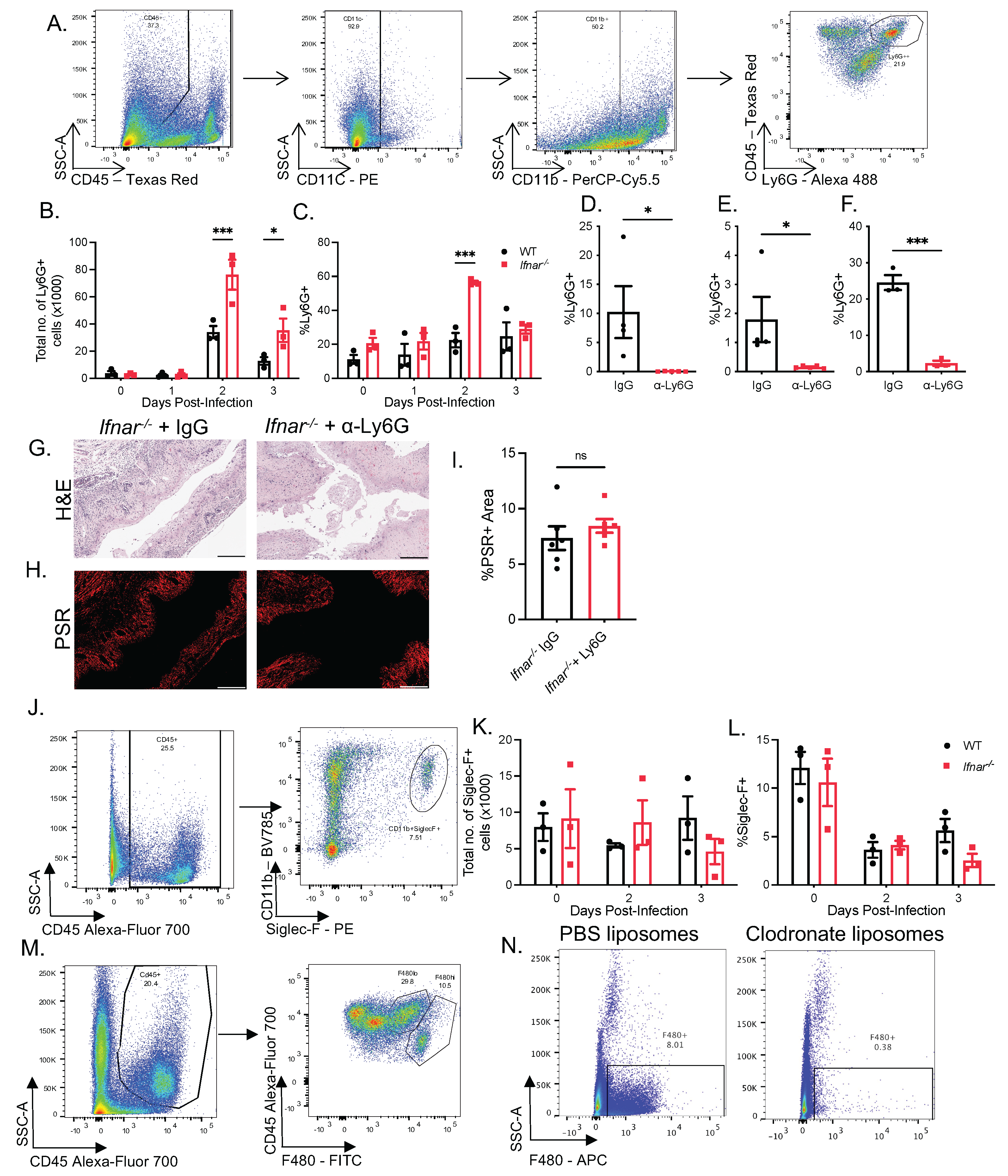

Supplement: S3 Fig — (A) Representative flow plots for gating CD45+CD11c-CD11b+Ly6G+ neutrophils in vaginal tissue of HSV-2-infected WT and Ifnar-/- mice. (B to C) Total number of neutrophils (B) and proportion of neutrophils to total CD45+ cells (C) in vaginal tissue of HSV-2-infected WT and Ifnar-/- mice at 0 to 3 dpi (n = 3). (D to F) Proportion of neutrophils to total CD45+ cells in blood (D; n = 4) spleen (E; n = 4) and vaginal tissue (F; n = 3) in HSV-2-infected WT mice at 3 dpi. (G) H&E staining of vaginal cross-sections of infected Ifnar-/- mice with isotype or α-Ly6G Ab at 3 dpi (n = 3). (H and I) PSR staining (H) and % PSR+ to total vaginal tissue area (I) of vaginal cross-sections of HSV-2-infected WT and Ifnar-/- mice with isotype or α-Ly6G Ab at 3 dpi. (J) Representative flow plots for gating CD45+CD11b+Siglec-F+ eosinophils in vaginal tissue of HSV-2-infected WT and Ifnar-/- mice. (K to L) Total number of eosinophils (K) and proportion of eosinophils to total CD45+ cells (L) in vaginal tissue of HSV-2-infected WT and Ifnar-/- mice at 0, 2 and 3 dpi (n = 3). (M) Representative flow plots for gating CD45+F480+ macrophages in vaginal tissue of HSV-2-infected WT and Ifnar-/- mice. (N) Representative flow plots for CD45+F480+ macrophages in vaginal tissue of HSV-2 Ifnar-/- mice administered PBS or clodronate liposomes at 3 dpi. Data in (B)–(F), (I), (K), (L) are represented as mean ± SEM. *p < 0.05, and ***p < 0.001 (B, C, K, L two-way ANOVA; D-F, I, one-way ANOVA). (TIF) [file ppat.1010471.s003.tif]

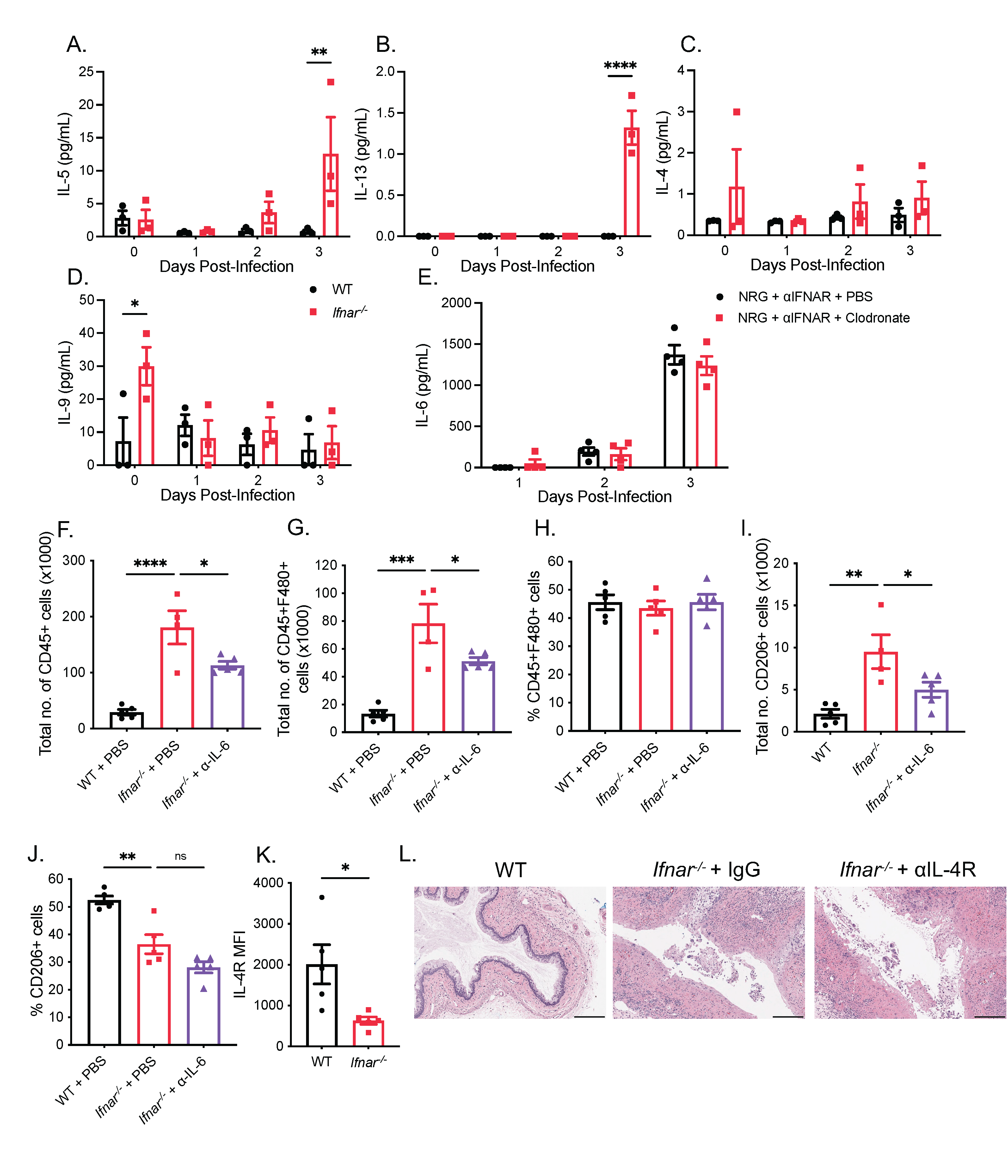

Supplement: S4 Fig — (A to D) IL-5 (A), IL-13 (B), IL-4 (C), and IL-9 (D) cytokine levels in vaginal washes of HSV-2-infected WT and Ifnar-/- mice at 0 to 3 dpi (n = 3). (E) Levels of IL-6 in vaginal washes of HSV-2-infected NRG α-IFNAR Ab with PBS or clodronate liposomes (n = 4). (F) Total number of CD45+ cells in the vaginal tissue of WT and Ifnar-/- mice administered PBS or α-IL-6 at 3 dpi (n = 4–5). (G—H) Total number of CD45+F480+ cells (G) and % CD45+F480+ to total CD45+ cells (H) in vaginal tissue of infected WT and Ifnar-/- mice administered PBS or α-IL-6 at 3 dpi (n = 4–5). (I-J) Total number CD45+F480+CD206+ cells (I) and % CD206+ to total CD45+F480+ cells (J) in vaginal tissue of infected WT and Ifnar-/- mice administered PBS or α-IL-6 at 3 dpi (n = 4–5). (K) IL-4R MFI of vaginal tissue CD45+F480+ cells of HSV-2-infected WT and Ifnar-/- mice at 3 dpi (n = 5). (L) H&E staining of vaginal cross-sections for HSV-2-infected WT and Ifnar-/- mice administered isotype of α-IL-4R at 3 dpi, scale bar represents 200 μM (n = 5). Data in (A-K) are represented as mean ± SEM. *p < 0.05, **p < 0.01, ***p < 0.001, and ****p < 0.0001 (A-E, two-way ANOVA; F-J, one-way ANOVA; K, two-tailed t-test). (TIF) [file ppat.1010471.s004.tif]

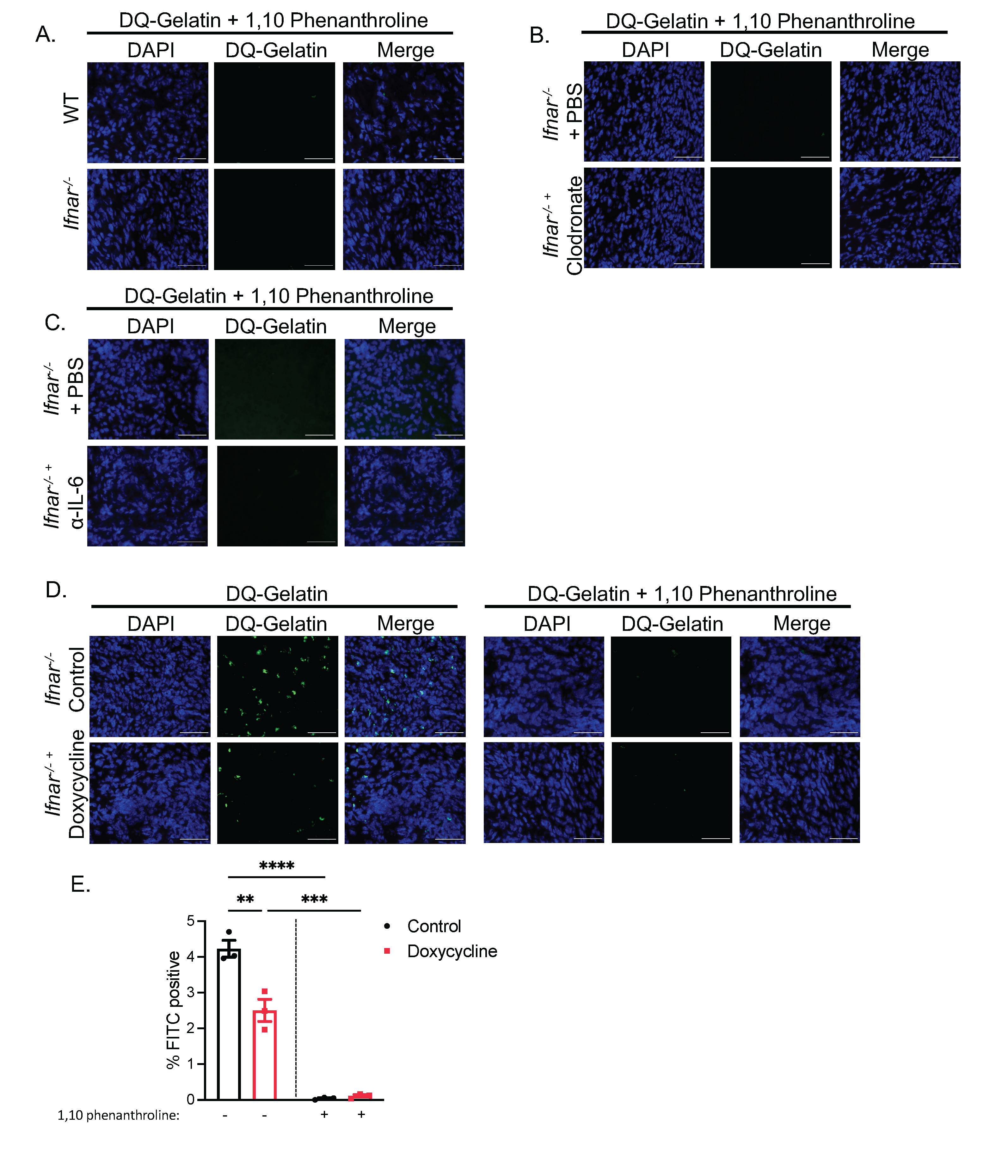

Supplement: S5 Fig — (A) Representative control images of in situ zymography submucosa with 1,10 phenanthroline of HSV-2-infected WT and Ifnar-/- mice at 2.5 dpi. (B) Representative control images of in situ zymography 1,10 phenanthroline of HSV-2-infected Ifnar-/- mice administered PBS or clodronate liposomes at 2.5 dpi. (C) Representative control images of in situ zymography 1,10 phenanthroline of HSV-2-infected Ifnar-/- mice administered PBS or α-IL-6 water at 2.5 dpi. (D) Representative images of in situ zymography submucosa of Ifnar-/- mice administered control or doxycycline in drinking water at 2.5 dpi with or without 1,10 phenanthroline control. (E) % FITC positive cells in (D) (n = 3). Data in (E) represented as mean ± SEM. **p < 0.01, ***p < 0.001, and ****p < 0.0001 (two-tailed t-test). (TIF) [file ppat.1010471.s005.tif]

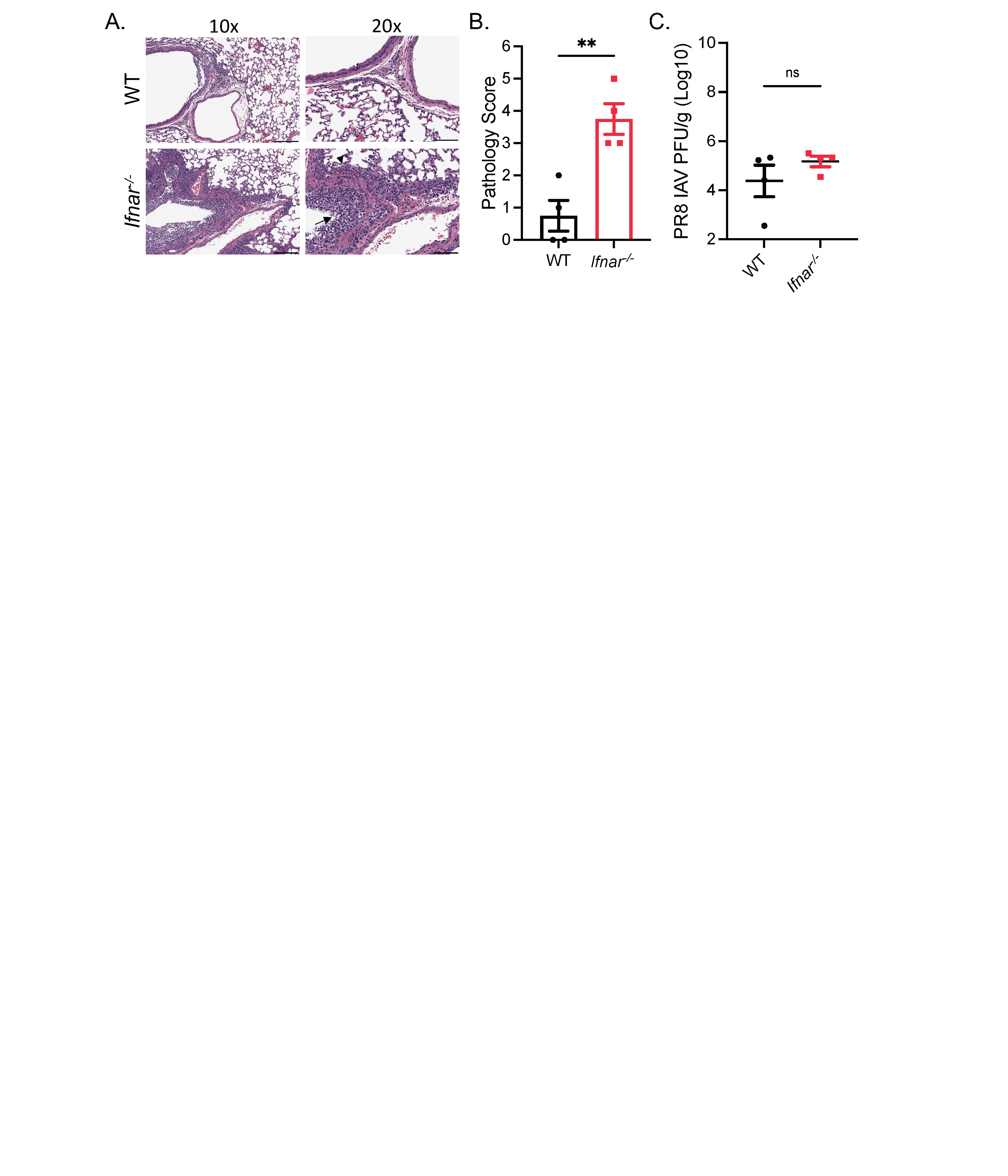

Supplement: S6 Fig — (A) H&E staining of lung cross-sections from WT and Ifnar-/- mice infected with 300 PFU IAV at 5 dpi. (n = 4). (B) Pathology score of (A). (C) Lung viral titers of mice in (A) at 5 dpi. Data in (B), (C) are represented as mean ± SEM. **p < 0.01 (two-tailed t-test). (TIF) [file ppat.1010471.s006.tif]

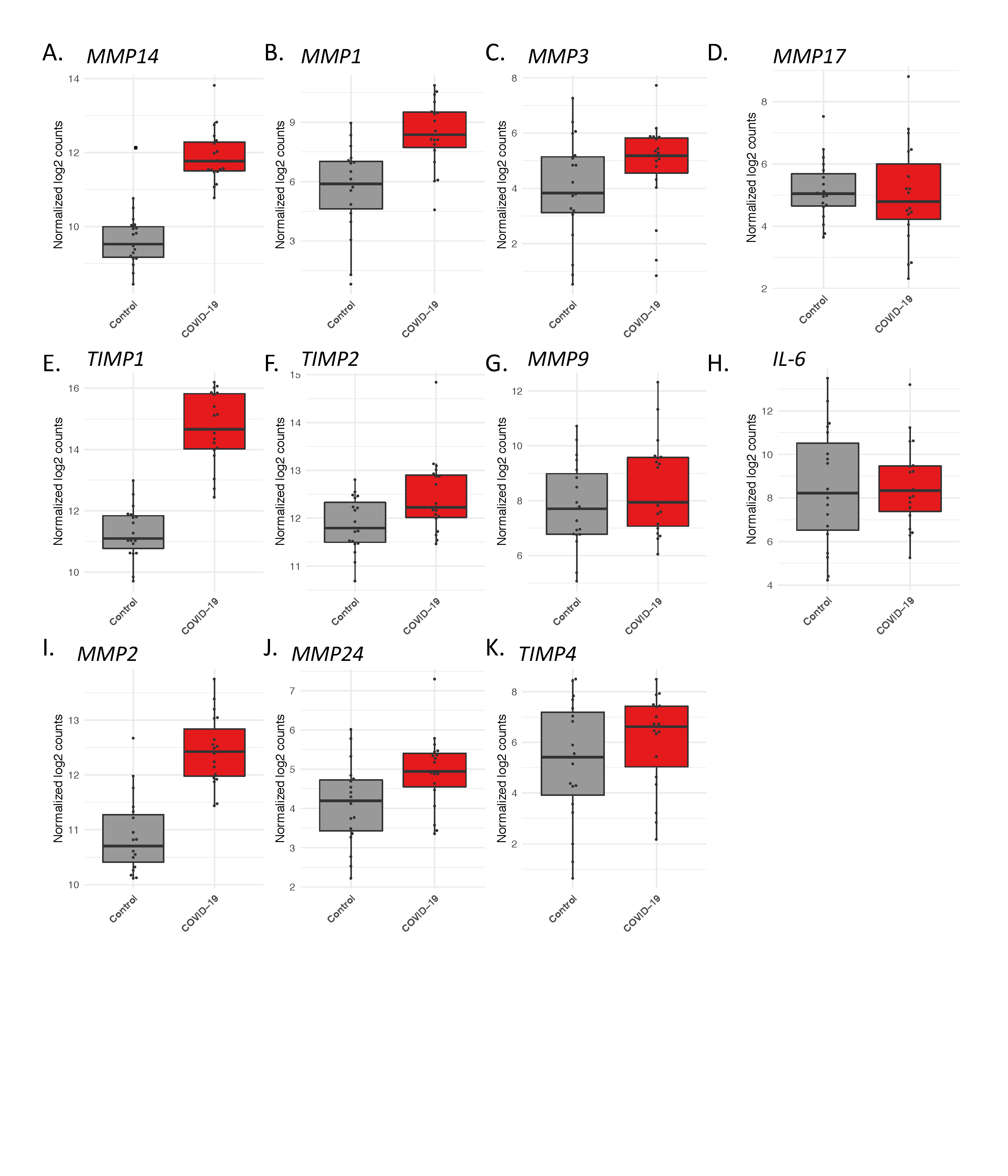

Supplement: S7 Fig — (A-K) Box plot of Nanostring log2 counts between control and COVID-19 patients for (A) MMP14 (B) MMP1 (C) MMP3 (D) MMP17 (E) TIMP1 (F) TIMP2 (G) MMP9 (H) IL-6 (I) MMP2 (J) MMP24 (K) TIMP4. (TIF) [file ppat.1010471.s007.tif]
