# Supplementary material for: Type I interferon regulates proteolysis by macrophages to prevent immunopathology following viral infection
Source: PLoS Pathog. 2022 May 5;18(5):e1010471. doi: 10.1371/journal.ppat.1010471 (PMC9113601; doi:10.1371/journal.ppat.1010471)
Supplement: S1 Table — (DOCX) [file ppat.1010471.s008.docx]

**S1 Table: Characteristic of the COVID-19 Patients in TMA**

|  |  | **COVID-19 Patients** |
| --- | --- | --- |
| **Number of Patients** |  | 8 |
|  |  |  |
| **Age (Years)** | **Range** | 54-91 |
|  | **Mean** | 74.9 |
|  |  |  |
| **Sex (#)** | **Female** | 3 |
|  | **Male** | 5 |
