# Supplementary material for: Type I interferon regulates proteolysis by macrophages to prevent immunopathology following viral infection
Source: PLoS Pathog. 2022 May 5;18(5):e1010471. doi: 10.1371/journal.ppat.1010471 (PMC9113601; doi:10.1371/journal.ppat.1010471)
Supplement: S2 Table — (DOCX) [file ppat.1010471.s009.docx]

**S2 Table. Characteristic of control and COVID-19 patients in gene expression analysis**

|  |  | **Control Patients** | **COVID-19 Patients** |
| --- | --- | --- | --- |
| **Number of Patients** |  | 19 | 19 |
|  |  |  |  |
| **Age (Years)** | **Range** | 0-83 | 54-96 |
|  | **Mean** | 35.4 | 76.7 |
|  |  |  |  |
| **Sex (#)** | **Female** | 6 | 4 |
|  | **Male** | 11 | 15 |
|  | **Undisclosed** | 2 | 0 |
