# Supplementary material for: Type I interferon regulates proteolysis by macrophages to prevent immunopathology following viral infection
Source: PLoS Pathog. 2022 May 5;18(5):e1010471. doi: 10.1371/journal.ppat.1010471 (PMC9113601; doi:10.1371/journal.ppat.1010471)
Supplement: Attachment — Submitted filename: Response to reviewers.docx [file ppat.1010471.s011.docx]

Point-by-point response to the reviewers’ comments

We would like to thank the reviewers and editors for taking the time to review our manuscript and providing their insightful and constructive comments. We took the comments to heart and revised the manuscript by adding new experimental data and providing further clarifications. Below is our detailed response to every single comment and how we revised the manuscript accordingly (responses are in blue font).

**Point-by-point response to reviewer#1**:

“Amanda Lee et al. demonstrated the key role of type 1 interferons in mucosal viral infections. The absence of type I IFN signaling is linked with several genital tissue destruction during HSV-2 infection. They demonstrated interesting findings in IFNAR-deficient mice linked with immunopathology. However, there are some clarifications needed related to the choice of inhibitors and the role of MMPs and active MMPs in virus infection.”

**Response**: We thank the reviewer for finding our work interesting in regard to the mechanisms of immunopathology. We also thank the reviewer for the detailed comments on the methodology and conclusions made in our manuscript.

**Comment 1** - “Line 254, 1,10 phenanthroline can inhibit MMPs but it is not a specific inhibitor. It is a broad spectrum and it should be changed to a “broad-spectrum metalloprotease inhibitor.” 1,10 phenanthroline acts via the removal and chelation of the metal ion required for catalytic activity. Therefore, it also inhibits other proteases that degrade the ECM and tissue remodelling such as the ADAMs and ADAMTSs metalloproteases. It can also inhibit bacterial and viral metalloproteases. Therefore, no conclusions to specific inhibition of MMPs can be drawn.”
**Response:** We thank the reviewer for this comment. The use of 1,10 phenanthroline in the *in situ* zymography assay was as a negative control to ensure the staining was not the result of nonspecific background staining. We agree that it is not a specific inhibitor but a chelator that broadly inhibits enzymatic activity. On line 367-370, we have changed the wording to “broad-spectrum metalloprotease inhibitor”.

**Comment 2** - “Line256, what are gelatinase-positive cells? Do they authors refer as MMP2 and MMP9 producing cells? If it is the case, it is a term that is no longer used and should be referred as MMP2 and MMP9. Also, Gelatin is denatured type IV collagen and is not naturally occurring in vivo and is an artifact of gel denaturation or frozen histology as performed by in situ zymography.”

**Response**: We thank the reviewer for bringing this point to our awareness. We agree that our intention is to describe them as MMP-2 and MMP-9 producing cells that are able to degrade DQ-gelatin provided in an *in situ* assay. We have changed their descriptions on line 367 to 375 to “MMP-2 and MMP-9 activity” and “MMP-2 and MMP-9 expressing cells”.

**Comment 3** - “Doxycycline has been demonstrated to inhibit MMP activity but again, it is not a specific MMP inhibitor. It can inhibit multiple other proteases including bacterial collagenases, among many others. Therefore, the conclusion (Line 263-264) that MMP production by macrophages plays a role in virus-induced vaginal immunopathology is not supported by the data. A more accurate statement would be that “metalloproteases” (from mice, bacteria or virus) can regulate this process. The same issue with Line 280-281. It should be changed to metalloproteases and not MMPs specifically.”

**Response:** We agree with the reviewer that doxycycline is not a specific MMP inhibitor but has rather broad specificity. We have changed the description to “administration of doxycycline, known to suppress a broad range of metalloproteases”. In addition, to strengthen our argument that MMPs are involved, we have included a new experiment, administering the MMP inhibitor RO 28-2653. This drug has high selectivity towards MMP-2, MMP-9, and MMP-14 (47). We show in panel J-L in figure 5 that administration of Ro 28-2653, similar to doxycycline, reduces the development of pathology without impacting viral clearance.

We have also added in the results section:

“To further confirm the role of MMPs in viral-induced pathology, we administered Ro 28-2653, an MMP inhibitor with preference towards MMP-2, -9, and MMP-14. Similar to doxycycline, Ro 28-2653 was able to reduce the inflammation and pathology in *Ifnar^-/-^* mice in response to HSV-2 infection, without affecting viral clearance (Figure 4J-L). These experiments suggest that MMP production by macrophages plays a key role in virus-induced vaginal immunopathology.”

**Comment 4** - “Figure 7, the authors show data that mRNA levels of MMP1, -2, -14 and -24 are elevated. An elevation of mRNA levels does not imply increased MMP activity. Also, TIMP1 and TIMP2 are also elevated. TIMPs are inhibitors of MMP activity. Therefore, there is no evidence on line 294-295 that the MMP activity is elevated in post-mortem COVID19 patients. To assess MMP activity, one would need to use activity-based probes that bind active MMPs. This conclusion cannot be supported by the data presented here. Same apply for Line 369-370. There is no evidence by these data that doxycycline would inhibit MMPs activity alone, without side effects, in addition that the authors did not demonstrate increased MMP activity but a change in mRNA levels.”
**Response**: We agree that mRNA levels do not necessarily correspond with increased MMP activity, and have thus changed the wording to “increased macrophage presence and MMP expression” in the results section on line 438. In the conclusion, we agree that we cannot concretely conclude that MMP activity is elevated in COVID-19 patients. However, we hypothesise based on the RNA-seq data and our findings of the mechanisms of pathology to mucosal infection, that inhibition of MMP activity may be an effective treatment strategy for COVID-19 patients and other severe viral infections, and our findings support further research into this avenue.

In addition, we do not think that an increase in TIMPs disproves our hypothesis that MMP activity may be increased in COVID-19 patients. While TIMPs are commonly known as inhibitors of MMPs, their relationship with MMPs can be quite complex. For example, TIMP-2 is also known to regulate and enhance MMP-2 and MMP-14 activation through the MMP-2/MMP-15 axis (Bernardo et al. Biochem J 2003). It is also commonly observed that TIMP-1/2 can be elevated in viral infection at the same time as MMPs, and it is more important to look at the ratio of MMP and TIMP activity in determining disease outcomes rather than TIMP levels in isolation (Lee et al. Virus Research 2013;Ulrich et al. J. Neuropathol. Exp. Neurol. 2006;Khuth et al. 2020 J Virol). Therefore, increased TIMP activity may not be sufficient to suppress pathogenic MMP activity.

Doxycycline is a commonly used antibiotic for treatment of bacterial infections, and in short doses has been demonstrated to be tolerable. Their toxicity, as well as toxicities of other MMP inhibitors has been specifically linked towards long term use as a treatment against chronic inflammatory diseases and cancer. In the case of COVID-19, treatment would be for acute infections, and has been used in COVID-19 patients to treat virus-induced bacterial pneumonia. Our findings suggest that doxycycline may also act to inhibit MMP-mediated tissue damage alongside its antibiotic functions.

With these comments, we amend our discussion to the following:

“Inhibition of MMP proteolytic activity offers an alternative and effective therapeutic target downstream of these pathways to suppress immunopathology to infection, without impairing viral clearance. Administration of doxycycline and Ro 28-2653 during HSV-2 and IAV infection substantially reduced the extent of tissue damage in separate mucosal tissues. Increased expression of MMPs in COVID-19 patients also suggests that MMP inhibitors could act as effective treatment strategy for severe infection in human patients. Currently, the administration of doxycycline in COVID-19 patients has been due to their antiviral properties, and prevention and treatment of secondary bacterial pneumonia (68,69). Their capacity to control disease severity of both bacterial-induced sepsis and influenza infection has also been attributed to their ability to inhibit mitochondrial protein synthesis (70). We recognise that as a limitation of the COVID-19 patient data, we cannot conclusively determine that our transcriptional data correlates with increased MMP activity as a cause of pathology due to COVID-19 infection. Our findings support further research into investigating the feasibility and efficacy of MMP inhibitors, such as doxycycline, in the treatment and prevention of viral-induced pathology, and further mechanisms regulating MMP function.

**Comment 5** - “MMPs do many more functions in virus infection other than ECM degradation. Examples include: 1- Marchant et al. (2014) Nature Medicine, 20(5) 493-502; 2- Cheung C, et al (2008) Circulation 117:1574–1582; 3- Yang S, et al. (2019) J Hepatol 71:685–698; 4- Zhang K, et al. (2003) Nature Neuroscience 6:1064–1071. 5- Vergote D, et al. (2006) Proc Natl Acad Sci 103:[19182–19187](tel:19182–19187). Therefore, the statement that MMPs degrade the matrix, ECM, and can result in tissue destruction is outdated. There are multiple other roles by MMPs during viral infection such as regulation and processing of chemokines, cytokines, cell surface receptors, etc.”

**Response**: We thank the reviewer for this comment and understand their viewpoint. While our description of MMPs in the discussion does include some of these functions, we agree that our statement in the results section may not be up to date. We have amended our description in the results section to the following:

“IL-6 has also been identified to induce macrophage production of MMPs, a class of calcium-dependent zinc-containing endopeptidases that degrade the extracellular matrix (ECM) (36,37). During viral infection, MMPs playing critical roles in tissue repair, cell migration, regulating cytokine and chemokine activity, and overall leukocyte recruitment during infection (38–40). Additionally, multiple MMPs, including MMP-2, MMP-9 and MMP-14, have been implicated in tissue pathology in the lung mucosa and female genital tract during infection (41–44).”

We have also expanded on the functions of MMPs in the discussion to the following:

“MMPs have also been shown to regulate immune cell recruitment to viral infection through endothelium ECM degradation to facilitate immune cell migration, as well as cleavage of chemokines and cytokines, thereby exacerbating inflammation and immune cell activation (38–40). Thus, the production of a variety of metalloproteases following severe viral infection not only weakens the structure of the basement membrane, but would also promote further mechanisms of immune-mediated inflammation.”

**Comment 6** – “Multiple MMPs can be produced by other immune cells such as neutrophils (MMP8, MMP9 and MT6-MMP), monocytes (multiple MMPs), T cells (multiple mmps), etc. It is true that there are increased recruitment of macrophages but how do the authors know that this effect is entirely linked to macrophages and not partially due by neutrophils, monocytes or T cells?”

**Response:** We agree that many immune cells, and in particular neutrophils and monocytes, are known as potent producers of MMPs. We believe this effect to be largely the response of macrophages, as we have demonstrated that neutrophils (via α-Ly6G depletion) and T cells (via the use of NRG mice) are not involved in the development of pathology. Therefore, their contribution to MMP production is not consequential to tissue pathology. In addition, previous studies by our research group and others have shown that *Ifnar^-/-^* mice show impaired recruitment of Ly6C^hi^+ monocytes to the tissue of infection, so they cannot be involved in tissue pathology (Lee et al JEM 2017).

**Comment 7** - “Abstract and overall conclusion, Line 43-44: the authors state that “uncover MMPs as therapeutic targets towards viral infections” is not supported by the inhibition data they demonstrated or the post-mortem COVID19 patients analysis.”

**Response**: To address this comment, we have included new data using the MMP-specific inhibitor Ro 28-2653. We hope the reviewer will agree with us that the new data sufficiently supports our hypothesis that MMPs are involved in the development of tissue pathology.

**Comment 8** – “Post-mortem information of COVID19 should be presented. What were their age and sex?”

**Response:** We agree with the reviewer and have added the patient data in Supplementary Table 1 and 2.

**S1 Table: Characteristic of the COVID-19 Patients in TMA**

|  |  | **COVID-19 Patients** |
| --- | --- | --- |
| **Number of Patients** |  | 8 |
|  |  |  |
| **Age (Years)** | **Range** | 54-91 |
|  | **Mean** | 74.9 |
|  |  |  |
| **Sex (#)** | **Female** | 3 |
|  | **Male** | 5 |

**S2 Table. Characteristic of control and COVID-19 patients in gene expression analysis**

|  |  | **Control Patients** | **COVID-19 Patients** |
| --- | --- | --- | --- |
| **Number of Patients** |  | 19 | 19 |
|  |  |  |  |
| **Age (Years)** | **Range** | 0-83 | 54-96 |
|  | **Mean** | 35.4 | 76.7 |
|  |  |  |  |
| **Sex (#)** | **Female** | 6 | 4 |
|  | **Male** | 11 | 15 |
|  | **Undisclosed** | 2 | 0 |

**Minor comments**:

“1-Line 40 and line 48 (and throughout the manuscript): As per Uniprot nomenclature, MMPs abbreviation should be changed to matrix metalloproteinases (MMPs)

2- Figure 5G: Doxycycline has a capital “O” that should be changed.

3- S5 fig, Line 531: the word “specific” should be removed as 1,10 phenanthroline is not a specific MMP inhibitor.

4- S7 Fig and Fig7, Line 544-544: Gene names should be italicized.”

**Response**: We thank the reviewer for pointing out these minor comments. We have changed the abbreviation to matrix metalloproteinases. In addition, the capital O has been changed on Figure 5G. We have also removed the word “specific” to “metalloproteinase inhibitor” for 1,10 phenanthroline. Finally, the gene names have been italicized on Figure 7 and S7.

**Point-by-point response to reviewer#2:**

“The manuscript submitted for review by Lee et al. shows how the absence of IFNAR is associated with immunopathology following viral infection. They use examples of overly active immune responses brought by fatal Ebola infection (discussed in the introduction), and experiments with HSV-2, SARS-CoV2 and Influenza A virus are presented. They discuss how early type I IFN is associated with disease tolerance by SARS-CoV2 whereas delayed or reduced type I IFN is associated with cytokine storms and severe disease. The impactful findings in this study are linking IL6 expression with retarded type I IFN to macrophage MMP expression. This is significant since out of control IL6 expression is associated with hemorrhagic viral disease and ARDs. IL6 has been thrust to the forefront during the COVID19 pandemic because it is seen by the community as one of the principle culprits in ARDS due to SARS-CoV2. I cannot find a paper like this one though there are papers here and there that tackle this area in a nebulous way. However no one has connected the mechanistic dots like this paper, that is, high IFN-I, viral control and low IL6 and macs-MMPs low path, but if low IFN-I leads to high IL6, high macs and MMPS and tissue destruction and mayhem.

In this substantial and compelling paper they use a number of different viruses and models. They start by exploring the intravaginal model of herpes simplex virus type -2 infection. They found that HSV-2 induced pathology was significantly worse in the reproductive mucosae of IFNAR KO female mice. They also used an anti-IFNAR neutralizing antibody in WT mice and showed the same extensive pathology due to HSV-2 infection. There was an increase in CD45+ cells in the mucosae of IFNAR KO mice and a significant decrease in collagen. Other experiments suggested that viral load was not responsible for the increased pathology.

Experiments in the IFNAR KO mice using IL12 and 18 to induce IFNgamma expression reduced HSV-2 mucosae pathology. Pathology was recapitulated with α-NK1.1 mAb treatment of IFNAR DKO mice, that reduced NK cells. Nor were ILC2 cells and Th2 cells involved in the immunopathology, using NRG mouse treated with the IFNAR monoclonal that induced the HSV-2 pathology in NRG mice. They also showed that neutrophils and eosinophils were not involved in the effect that led them to macrophages and somehow, MMPs in influenza mouse model and COVID infected samples.

Assessment and major criticism. The authors show that type I interferon keeps MMP secretion by macrophages at bay. Otherwise there is extensive tissue damage by influx of macrophages secreting gelatinases and other MMPs. The work is well done, with extensive controls, and the manuscript is well written and easy to follow. The data are interesting.

The link between IL6 expression and macrophages during viral infection is likely the most significant of the many discoveries presented by this study. The last section of the paper is where the authors pivot to MMPs but there is no reasoning as to why focus on these proteases. This comes down to improving/providing a lead up rationale for studying MMPs. Superficially, one might argue they used rather blunt tools to inhibit MMPs which I think is the biggest drawback to the paper if it can be considered a drawback. They used 1,10-Phenanthroline and doxycycline both of which have other minor side effects. For example, 1,10-Phenanthroline is a Zn chelator and plucks the Zn out of MMP active sites and possibly other Zn dependent mechanisms. However I don’t see any of these impacting their experimental conclusions owing to the numerous controls. I should add that the authors’ use of these MMP inhibitors was well controlled (doxycycline didn’t affect viral load but reduced epithelial mucosae integrity) and these inhibitors are established in the field with well characterized profiles. The problem with MMPs is that their active sites share very high identity and thus it is very difficult-near impossible to develop an MMP specific inhibitor. With even the most specific MMP inhibitors there is significant cross over inhibition to other MMPs.

The authors used what might be considered by the MMP neophyte as rather blunt instruments to study the involvement of MMPs. The case could be made for further experiments using the various MMP KO mice that are available but this would show them what they already know. It might help identify an MMP(s) responsible for HSV-2 pathology though many papers have been published trying to make a case for one MMP responsible for a given disease but these papers are rarely convincing and usually incorrect. Tissue destruction is due to the expression of a range of MMPs. Therefore I don’t think that further use of MMP KO mice would provide much more insight or change the conclusions of the manuscript. The final figure that looked at MMP expression in COVID patients opens up the topic nicely for further studies to look at the individual MMPs and their substrates in viral infections by others.”

**Response**: We thank the reviewer for finding our manuscript of interest with compelling data important to the field of viral immunology. We also thank the reviewer for their thoughtful and careful analysis of our findings, and assessment of our methods and controls. We carefully considered all their comments and advised the manuscript accordingly below.

**Comment 1** – “Abstract. The second sentence is awkward and not entirely true. IFN type I can make one more susceptible to Lupus for example. I would change the wording from, “less is known about how IFNs prevent immunopathology and increase disease tolerance,” to something like “…less is known about how IFNs affect immunopathology and related disease.”

**Response:** We agree with the reviewer and appreciate their suggestion. We have changed the second sentence of the abstract to the following:

“While the role of type I interferons (IFNs) in early control of viral replication is clear, less is known about how IFNs can regulate the development of immunopathology and affect disease outcomes”

**Comment 2** – “Introduction. I suggest being more focused in the introduction of the paper. There is discussion of influenza, Ebola and SARS-CoV2 but no introduction to HSV-2, a very different virus in its own right. One may mention these other viruses but I strongly suggest focusing the introduction on HSV-2 since this is the research topic.”

**Response**: We thank the reviewer for this comment. To address this comment, we have revised the first two paragraphs of the introduction to the following:

“Effective early innate immune responses are crucial in determining the clinical outcomes of viral infections. Dysregulated inflammatory immune responses will not only impede early viral replication, but ultimately lead to immune-mediated tissue pathology. Type I interferons (IFNs) are master regulators of the early innate immune response and determinants of disease outcome against viral infection (1,2). For instance, during genital herpes simplex virus type 2 (HSV-2) infections, type I IFN induction stimulates early antiviral immunity through promoting monocyte recruitment and Natural Killer (NK) cell production of IFN-γ, necessary for host defense (3). Absence of type I IFN signaling results in increased viral replication and reduced survival to vaginal HSV-2 infection (3,4).

Studies of highly pathogenic viral infections including severe acute respiratory syndrome coronavirus type 2 (SARS-CoV)-2 and Ebola virus (EBOV) demonstrate that early type I IFN induction consistently correlates with disease tolerance (5–7).These immunopathogenic responses often occur independently of viral load, and are the result of dysregulated heightened inflammation (8). Type I IFNs have demonstrated an ability to control potentially pathogenic immune responses. During HSV-2 infection, type I IFNs both induce IFN-γ production by NK cells, but also inhibit their production during later stages of infection (9). In a mouse model of influenza infection, type I IFNs have also been demonstrated to inhibit various sources of immune-mediated pathology (10,11). Understanding how type I IFNs can both promote viral clearance, but also control inflammation to improve disease outcomes, is critical to understanding the mechanisms of disease pathology and develop effective treatments for viral infections.”

**Comment 3** – “Line 120. They need a lead up in this paragraph of the results section to explain what IL15 does and why they looked at IL15 DKO mice. This will make the paper easier to read as the rationale at this point in the paper is not clear.”

**Response**: We thank the reviewer for this suggestion to improve the clarity of our manuscript. We have amended the results section to the following:

“Loss of IFNAR can result in failure to control viral replication (3,27,28). To determine if an increase in viral replication was responsible for mucosal tissue pathology observed in *Ifnar^-/-^* mice, we compared HSV-2 infection in *Ifnar^-/-^* and *Il15^-/-^* mice. Loss of IL-15 during viral infection impairs NK cell maturation and innate IFN-γ production, resulting in increased HSV-2 viral titers compared to WT mice (28).”

**Comment 4** – “Line 247. There is no lead up explanation as to why the authors chose to look at MMPs so this needs to be corrected in a revision. There are other proteases secreted by macrophages so why MMPs? This is of course rhetorical in that MMPs are some of the best characterized proteases in immunity. Though it is not for me to write the paper but the authors can provide their own reasoning in a lead up paragraph. I suggest citing the relevant literature as there are a few papers already looking at mechanisms of MMPs during viral infection.”

**Response**: We thank the reviewer for this comment and agree that a discussion on the decision to study MMPs would improve the clarity and flow of the manuscript. We have added to our results section the following:

“IL-6 has also been identified to induce macrophage production of MMPs, a class of calcium-dependent zinc-containing endopeptidases that degrade the extracellular matrix (ECM) of the basement membrane (36,37). During viral infection, MMPs playing critical roles in tissue repair, cell migration, regulating cytokine and chemokine activity, and overall leukocyte recruitment during infection (38–40). Additionally, multiple MMPs, including MMP-2, MMP-9 and MMP-14, have been implicated in tissue pathology in the lung mucosa and female genital tract during infection through mediating inflammation and ECM degradation (41–44).”

**Comment 5** – “The figures are neat and compelling but the font is a touch small and fuzzy in a lot of the panels. I suggest increasing the font size in these figures to make the figures easier to read. The HandE and other tissue stains are also fuzzy and impossible to interpret. The authors have done themselves no favors (or the reviewer) in submitting figures like this which I find quite aggravating. The figures all need to be fixed in this regard.”

**Response**: We thank the reviewer for finding the figures neatly prepared. We agree with the reviewer’s comment and have increased the font sizes in the figures. We have increased the resolution of the figures to the maximum file size allowed by PLOS Pathogens.

**Comment 6** – “Materials and methods. This section appears to be the most lacking in detail. I don’t believe there is a size limit for PLoS Pathogens so the authors can put significantly more work into this section rather than merely citing previous papers.”

**Response**: We have included more details in the materials and methods section where it was not sufficiently explained. This includes increased detail on the immunoblot analysis protocol.

**Point-by-point response to reviewer#3:**

“The manuscript “Type I interferon regulates proteolysis by macrophages to prevent immunopathology following viral infection” by Lee et al., describes prevention of immunopathology after mucosal virus infections by type I IFN. Using IFNAR knockout mice and two infection models, vaginal HSV-2 infection and intranasal infection with influenza virus, they show an IFNAR-dependent control of virus-induced pathology. Knockout of IFNAR results in immunopathology in local tissues after infection which can be prevented by depletion of macrophages and blocking of IL-6 signaling and also by substitution with IFNG. Additionally, they show that the observed immunopathology/tissue damage is promoted by metalloproteinases.
Although the finding that IFNs do regulate immunopathology and metalloproteinases are not new, this comprehensive analysis of the role of type I interferon in immunopathology using two virus infection models in the mouse is a novel and interesting approach. It brings together immunopathology observed after IFN-knockout and/or infection, a correlation of high IL6 levels and immunopathology, and abundant metalloproteinase expression associated with immunopathology. A major weakness of the studies is, that the authors claim or disclaim connections of these pathways without presenting convincing data confirming or disconfirming the connections.”

**Response:** We thank the reviewer for finding our manuscript a comprehensive analysis of the role of type I IFNs in virus induced immunopathology using two different viruses and two different mucosal sites. We have revised the manuscript and added new experimental data, as well as modified the text, to better support the main conclusions of our study.

**General comment**: “Although it is nowadays highly fashionable to connect any finding in the lung or in virus infections to SARS-CoV-2 infections, here, it does not strengthen the findings of this paper, especially as the connection is completely based on similarities in phenotypes and not on functional correlates. The SARS-CoV-2 connection regarding for example the treatment with doxycycline should be restricted to the discussion and the data removed.”
**Response:** We agree that during the COVID-19 pandemic, many studies tried to connect to lung viral infections and SARS-CoV-2. The main focus of this study was the role of type I IFNs in the regulation of the immune response during viral infection at mucosal sites, including lung mucosa. We agree that we don’t have substantial mechanistic human data, but we have included the human data that was relevant to the main conclusions that we drew from the murine models to support further investigation. In addition, reviewer #2 was of the opinion that: “The final figure that looked at MMP expression in COVID patients opens up the topic nicely for further studies to look at the individual MMPs and their substrates in viral infections by others.” To address the reviewer’s concerns, we have included in the discussion the limitations of the human data:

“We recognise that as a limitation of the COVID-19 patient data, we cannot conclusively determine that our transcriptional data correlates with increased MMP activity as a cause of pathology due to COVID-19 infection. Our findings support further research into investigating the feasibility and efficacy of MMP inhibitors such as doxycycline in the treatment and prevention of viral-induced pathology, and further mechanisms regulating MMP function.”

Major points:

**Comment 1** - “Induction of IFNG abolishes virus-induced immunopathology in IFNAR-knockout mice. The authors’ point that IFNA and IFNG-dependent signaling are independent, but redundant pathways suppressing virus-induced pathology is not convincingly shown. It is known that IFNG can upregulate IL-6 and that I can affect macrophage activity. IFNG is a known inducer of metalloproteinases. The authors present no convincing experiment which excludes that the IFNA-dependent protection from immunopathology is not due to downregulation of IFNG.”

**Response**: We thank the reviewer for this insightful point that type I and II interferons do not necessarily signal through independent pathways. We agree that absence of IFNAR leads to a lack of IFN-γ production following viral infection. However, the absence of IFN-γ at early stages of infection in the *Ifnar^-/-^* model is not a sufficient cause of tissue pathology, as IL-15^-/-^ mice that lack NK cells, and thereby early IFN-γ production, have similar viral replication but do not develop pathology following HSV-2 infection due to intact type I IFN signaling. While IFN-γ has been shown to upregulate IL-6, it has also been shown to counteract IL-6 signaling as well (Bluyssen et al Am. J. Physiol 2010). In addition, as described in Figure S1C, rescue of IFN-γ production via administration of IL-12/18 in *Ifnar^-/-^* mice, rescues pathology. We also show in Figure S1B and by Lee et al. Front. Immunol 2019, that wild-type mice with intact type I IFN signaling show significant production of IFN-γ 2 days post-infection by NK cells during HSV-2 infection, but no pathology. Thus, it could be said that pathology in the absence of type I IFNs could be the result of lack of IFN-γ signalling, but absence of IFN-γ alone is not sufficient for the development of pathology. This suggests that presence of either type I or II interferons at early infection is sufficient to suppress viral induced pathology. We have clarified our argument in the manuscript to the following:

“Administration of IL-12 and IL-18 induced IFN-γ production at 2 and 3 dpi in *Ifnar^-/-^* mice, rescued vaginal immunopathology and collagen staining, and reduced viral titers (S1C-1F Fig). This effect was dependent on NK cells, as depletion with α-NK1.1 mAb reversed the protection afforded by IL-12 and IL-18 administration in *Ifnar^-/-^* mice (S1C Fig). This suggests that the induction of type II IFNs is sufficient to prevent the development of viral-induced pathology in the absence of type I IFN signaling. Meanwhile, *Il15^-/-^* mice that possess no early IFN-γ production, but intact type I IFN signaling, show no development of tissue pathology. These results suggest that innate IFN-γ signaling and type I IFN signaling possess redundant roles in suppressing virus-induced pathology, as presence of either type I or type II IFN signaling is sufficient to prevent immunopathology.”

**Comment 2 -** “Figure 5: The authors claim that Figure 5 shows that type I IFN signaling suppresses IL-6 mediated MMP degradation of tissue. The figure only shows that macrophages mediate this degradation without showing for example the effect of anti-IL6 antibodies as used in figure 4. This has to be added to make their point.”

**Response**: We agree with the reviewer that the effect of anti-IL-6 on MMP activity is necessary to support our conclusion. To address this comment, we have performed new experiments and have added new experimental data on panel E and F on Figure 5 that shows that blockade of IL-6 in *Ifnar^-/-^* mice lead to reduced numbers of MMP-2 and MMP-9 producing cells, as shown below.

We have also added in the results section the corresponding text:

“In addition, we determined whether IL-6 was responsible for inducing MMP production from macrophages through administration of α-IL-6 in *Ifnar^-/-^* mice. α-IL-6 treated mice showed reduction in MMP-2 and MMP-9 producing cells compared to control mice (Fig 5E, 5F, and S5C Fig).”

**Comment 3 –** “In their second model, intranasal IAV infection, they claim to see the same effects in IFNAR knockout mice as they saw in vaginal HSV2 infection. Here, they only show IL6- dependency of MMP-dependent tissue damage. To make their point both in Figures 4 and 5, clodronate and anti-IL6 inhibition have to be shown.”

**Response**: We agree with the reviewer that depletion of macrophages with clodronate would be a logical following experiment. However, as stated by the supplier (https://clodronateliposomes.com/about-clodronate-liposomes/administration-protocols/), while clodronate administration can deplete macrophages from multiple sites in the body, it is unable to deplete macrophages in tissues such as the lung and brain. We have performed several experiments in our lab that systemic administration of clodronate does not lead to depletion of macrophages in the lung tissue, while intranasal delivery of clodronate only leads to depletion of alveolar macrophages. Many other labs have attempted to deplete macrophages in the lung tissue and have also been unsuccessful. Therefore, it is not possible to deplete lung macrophages to further establish our pathway in the IAV model.

Reviewer #3: Minor points:

**Comment 4** – “Using IL15 knockout mice to proof that elevated titers in HSV2 are not inducing immune pathology is not really suitable for showing that the immunopathology is not driven by elevated titers. IL15 knockout mice show differences in innate immunity, which are not defined here. Showing the IFNAR knockout plus clodronate (Figure 3 F) should be sufficient to make this point.”

**Response**: We thank the reviewer for the comment. We have decided to keep this figure, as the use of the IL-15^-/-^ further support our hypothesis of the redundant functions of type I and II IFNs during viral infection. We have also included in our results section a more detailed reasoning of our use of IL-15^-/-^ mice below:

“Loss of IFNAR can result in failure to control viral replication (3,27,28). To determine if an increase in viral replication was responsible for mucosal tissue pathology observed in *Ifnar^-/-^* mice, we compared HSV-2 infection in *Ifnar^-/-^* and *Il15^-/-^* mice. Loss of IL-15 during viral infection impairs NK cell maturation and innate IFN-γ production, resulting in increased HSV-2 viral titers compared to WT mice (28).”

**Comment 5 –** “Figure 4: Why should anti-IFNGR antibodies affect pathology in IL6 knockout mice if IL6 is a major downstream player in IFGNR knockout mice immunopathology? As to be expected, the effects were minor.”

**Response**: Figure 4 is using anti-IFNAR antibodies against the type I IFN receptor in the IL-6^-/-^ mice. The anti-IFNAR is necessary as in WT mice alone, there is not sufficient IL-6 production to induce IL-6-mediated pathology. Absence of type I IFN results in heighted IL-6 production necessary for the development of immunopathology. This experiment was a follow up control experiment to ensure that the anti-IL-6 antibody used in the *Ifnar^-/-^* mice was not acting non-specifically.

**Comment 6** – “Discussion: In the discussion the authors claim that type I IFN-dependent inhibition of MMPs is a novel finding. This is not true. There are numerous publications e.g. Sanceau et al., 2002 in Mechanisms of Signal Transduction that type I IFN does inhibit metalloproteinases. Surprisingly, none of them was cited by the authors. Selected citations should be added and discussed.”

**Response**: We thank the reviewer for this comment, and have included this short excerpt in the discussion:

“While MMP activity can be critical for tissue repair following IAV infection (57), excessive proteolytic breakdown of the ECM has been described in virus-induced pathology (40,43). Previous studies have observed type I IFNs inhibit TNF-α-dependent MMP-9 activation in tumour cell lines, and IFN therapy can suppress MMP expression in tumour models and during respiratory syncytial virus infection in mice (60–62). We observed that type I IFNs suppressed IL-6 and macrophage-dependent MMP production following mucosal viral infection.”

**Comment 7-** “In contrast to the description in the text, Fig S1F Fig 1L show identical data sets.”

**Response**: We thank the reviewer for their close attention to our figures. We have fixed the data set as shown below, to show the viral titres of *Ifnar^-/-^* mice given IL-12/18.

**Comment 8** - “Many figure numbers in the text are mixed up and are additionally not systematically organized (e.g. Fig. 3).”

**Response**: We agree with the reviewer and have fixed all the figure numbers. In addition, we have moved figure 3I to supplementary figure 3E such that the figures are in the order that they appear in the text.

**Comment 9 –** “Using a mouse virus instead of only human viruses adapted to mouse would increase the quality of the data and should at least be discussed.”

**Response**: We appreciate and thank the reviewer for this viewpoint. The majority of experiments in this study used HSV-2 333, which naturally infects many different specials of mammals, including humans and mice, although the disease manifestations are slightly different. While PR8 IAV is a mouse-adapted human virus, we have used this model as it is well established in the field as a useful experimental model of human influenza infection. We believe that a discussion regarding the use of viruses in the manuscript would not significantly alter the interpretation of the findings.

**Comment 10 –** “A figure depicting their model of the IFN-IL6-macrophage-metalloproteinase network would have been helpful.”

**Response**: We agree with the reviewer that such a figure would strengthen the manuscript. To address this comment, we have included figure 8 (below) that graphically summarizes our findings.
